# Keepin’ it real: Linguistic models of authenticity judgments for artificially generated rap lyrics

**DOI:** 10.1371/journal.pone.0224152

**Published:** 2019-10-22

**Authors:** Folgert Karsdorp, Enrique Manjavacas, Mike Kestemont

**Affiliations:** 1 Meertens Institute, Royal Netherlands Academy of Arts and Sciences, Amsterdam, The Netherlands; 2 Department of Literature, University of Antwerp, Antwerp, Belgium; The University of Memphis, UNITED STATES

## Abstract

Through advances in neural language modeling, it has become possible to generate artificial texts in a variety of genres and styles. While the semantic coherence of such texts should not be over-estimated, the grammatical correctness and stylistic qualities of these artificial texts are at times remarkably convincing. In this paper, we report a study into crowd-sourced authenticity judgments for such artificially generated texts. As a case study, we have turned to rap lyrics, an established sub-genre of present-day popular music, known for its explicit content and unique rhythmical delivery of lyrics. The empirical basis of our study is an experiment carried out in the context of a large, mainstream contemporary music festival in the Netherlands. Apart from more generic factors, we model a diverse set of linguistic characteristics of the input that might have functioned as authenticity cues. It is shown that participants are only marginally capable of distinguishing between authentic and generated materials. By scrutinizing the linguistic features that influence the participants’ authenticity judgments, it is shown that linguistic properties such as ‘syntactic complexity’, ‘lexical diversity’ and ‘rhyme density’ add to the user’s perception of texts being authentic. This research contributes to the improvement of the quality and credibility of generated text. Additionally, it enhances our understanding of the perception of authentic and artificial art.

## Introduction

Due to recent advances in computer technology, communication—be it verbal or not—is no longer a privileged kind of interaction that can only take place between human agents. Increasingly, people interact with a variety of artificial agents, sometimes even without being fully aware of whether or not their conversation partners are in fact human. Chatbot interfaces for customers at company websites are but one example of a popular application of this technology that is rapidly gaining popularity [1]. Across various domains, Natural Language Generation is increasingly high on the international research agenda [2]. Through advances in neural language modeling, it has become possible to generate synthetic texts in a variety of genres and styles [3]. While the semantic coherence of such texts should not be over-estimated, the grammatical correctness and stylistic qualities of these artificial texts are at times remarkably convincing.

In spite of the impressive advances in the field of natural language generation, however, the evaluation of such generative models remains a thorny issue—the same goes for generative models in other domains [1, 4]. This is especially true for texts produced in more specific artistic domains, such as literature or lyrics, where evaluative procedures must cover much more than basic criteria, such as grammatical correctness [5, 6]. In this paper, we focus on the issue of *authenticity*, i.e. a system’s ability to conceal its artificial nature. In the spirit of the seminal test proposed by [7], scholars nowadays generally agree that various factors relating to a system’s ‘Humanity’ have a profound impact on the (perceived) quality of a text synthesis system [8].

In this study, we report on a large-scale experiment in which we have crowd-sourced authenticity judgments for synthesized text at a major, mainstream music festival in the Netherlands. As a case study, we have turned to rap lyrics, an established sub-genre of present-day popular music [9–13] that is known for its typical textual and rhythmical properties [14–19]. We chose the specific application domain of rap lyrics, not only because this text variety would appeal to festival goers, but also because it is rich enough in constraints related to themes and style to make it a challenging domain for state of the art text generators. With its focus on rap lyrics, the current study adds to a growing body of research on computational creativity, which, amongst others, explores computational means to generate poetry [20], song lyrics [5], and literature [21].

Through a statistical analyses of the participants’ responses, we aim to advance our insight into the linguistic qualities that set generated lyrics apart from authentic ones. We build upon a handful of studies that chart linguistic differences between artificial and authentic text [22, 23]. Crucially, our focus on the analysis of authenticity cues adds a novel perspective to the study of the differential characteristics of authentic and artificial text. Research into the perception of generated text has been gaining momentum in recent years, in particular in the field of computational journalism—sometimes referred to as robot journalism [24, 25]. Studies such as [26], [27, 28] and [29], for example, analyze how news consumers perceive the quality, likability and credibility of artificially generated news. Results show little evidence for pronounced perception differences in readers’ assessment of the expertise and credibility of authentic and computer-written material. However, experimental results show that artificially generated materials are often rated slightly higher in terms of quality than authentic materials. As suggested by [26], this difference may be explained by subjects’ initial expectations of computer-generated text, and, conversely, high expectations of authentic text. If participants are disappointed in the quality of an authentic text, this may lead to enlarged negative assessment. If, on the other hand, a generated text positively surprises the reader, it can just as well lead to an overly positive assessment [29].

In addition to its focus on a different and relatively unexplored domain, our study distinguishes itself from the aforementioned text generation perception studies by scrutinizing specific linguistic properties distinctive of artificial and authentic texts. We investigate which linguistic features objectively distinguish original from generated text, and, subsequently, compare the results to those features that contribute to the participants’ *perceived* authenticity. As such, we aim to advance our understanding of the users’ perception to artificially generated content. More generally, we aim to contribute to the emergent field of natural language generation and suggest directions for the further improvement of both the quality and credibility of text generation systems. In particular, we address the following four research questions:

RQ1 To what extent can participants correctly discriminate between original and generated lyrics, i.e. *objective authenticity*.
RQ2 How is the authenticity of rap lyrics *perceived* by the participants, i.e. irrespective of whether their answer is objectively correct?
RQ3 Does the participants’ authenticity detection performance vary across different language generation architectures?
RQ4 Which textual and linguistic features distinguish authentic from generated text, either objectively or subjectively?

The remainder of this paper is structured as follows. First, we describe the experimental design of a serious game, which forms the empirical basis of our analyses. We then continue with a description of the data set of rap lyrics which we have mined for this study. We detail the relevant pre-processing steps and describe the ways in which we have enriched the raw material with additional linguistic information. We go on to offer a more formal description of the various text generation algorithms used for this study. We continue with describing a series of empirical models that capture the different linguistic characteristics of authentic versus generated rap lyrics. In the main section of this paper, we statistically analyze the crowd-sourced authenticity judgments. We place a firm emphasis on the linguistic properties of rap lyrics that seem to have served as authenticity cues for the participants. Finally, after discussing our results, we conclude the paper with a discussion and suggestions for future research. All code and data necessary to reproduce our research are available as supplementary material under a creative commons licence. See https://doi.org/10.5281/zenodo.3373757 for the web application and text generation models used in the experiment. See https://doi.org/10.5281/zenodo.3373648 for the code and data used to analyze the judgment data.

## Methods

### Experimental setup

**Fig 1:**
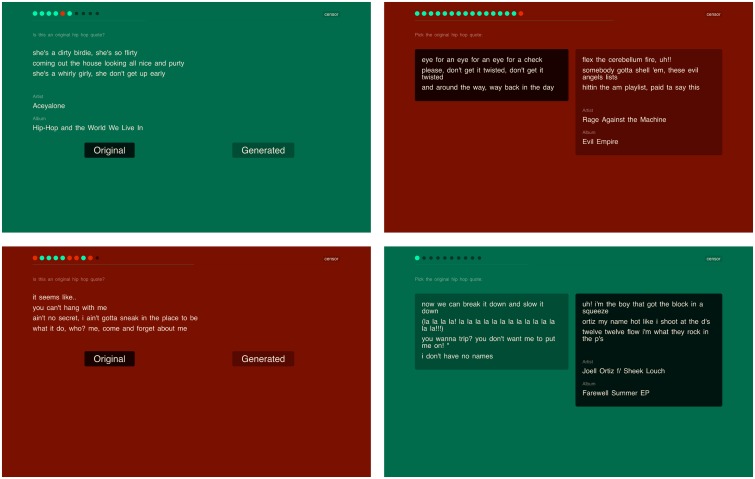
Illustrations (screenshots) of the interface. Illustrations (screenshots) of the interface. From top left to bottom right: (i) a correct answer to a type-B question, an incorrect answer to a type-A question, (iii) an incorrect answer to a type-B questions, and (iv) a correct answer to a type-A question. Similar visual feedback is provided in both situations: (1) the number of (in)correct answers is shown in the top (green and red circles); (2) immediately below, the progress bar shows the time elapsed; (3) provenance information (artist name and album title) appear below the authentic fragment.

The experiment took the form of an online serious game, which was played independently by individual participants. Participants were briefly explained what the game was about and how it should be played. Each game consisted of at least eleven questions (see below). After the first 10 questions, the game would switch into a ‘sudden death’ mode, allowing the player to continue until a false answer was given. To guarantee a smooth progression of participants, each question had to be answered within a reasonable time limit of 15 seconds, which was graphically displayed by means of a progress bar on screen. The interface (see Fig 1) would provide visual feedback as to the correctness of an answer immediately after it had been entered (the screen would turn red for incorrect answers and green for correct answers). Once a question has been answered, the interface would briefly display the original artist and song title for the authentic lyrics. The player was awarded one point for each correct answer given within the time limit. After completing the game, participants were presented their total score, and whether their score belonged to the top 10 best players.

#### Context

The experiment was conducted at the Lowlands Music festival in August 2018. It was carried out over a three-day period at the festival’s ‘Science park’, an enclosed area on the festival grounds dedicated to science, marked by clear signposts at the entrance of the zone. The experiment took place in a booth with three independent stations where the game could be played. Each station was supervised by a project collaborator/scientist, who was identifiable as such, because they wore a conspicuous ‘Lowlands Science’ shirt. The collaborators would concisely brief interested participants visiting the booth about the rules and (scientific) purpose of the game. Prior to the start of each game, verbal informed consent to participate in the gamified experiment was obtained. Minors were not allowed to enter the festival grounds, unless they were accompanied by an adult guardian. Other than this, no more specific demographics are available about the participants in the experiment, and no personal or identifying information were collected from the participants, since all data collected were strictly anonymous. The University of Antwerp Ethical Committee has assessed the objective and methodology of our study and confirmed that the study has been carried out in line with the required ethical standards. As such, the requirement for ethical approval was waived by the committee.

All participants were visitors of the music festival. In order to recruit a large and diverse sample of participants, the game was kept short and no personal information of the participants was recorded. A total of 701 participants played the game. Overall, 12 runs had to be excluded from analysis, because these runs were not completed. The stimuli in the experiment were fragments of original, authentic rap lyrics from the OHHLA corpus (detailed below) and synthetic fragments generated by one of the six artificial language models (also described below). These fragments were presented to the participants in the form of two types of questions (which were randomly alternated):

A The participants were simultaneously presented with an authentic fragment and an artificially generated fragment of rap lyrics, in written form on opposite sides (i.e. left and right) of a computer screen. The user had to decide which fragment was the authentic one. The positioning of the authentic and generated fragments alternated randomly.
B The participants were presented with a single fragment of rap lyrics, in written form on a computer screen. The user had to decide whether the fragment was authentic or computer-generated.

There are two important differences between both question types. In (A), participants typically had to process relatively more text than in (B), because there were two fragments involved and the allowed time for solving the question was the same in (A) and (B). One might hypothesize that this would render (B) easier than (A) in terms of cognitive processing time. On the other hand, (B) only presented participants with a single fragment, meaning that users were unable to explicitly compare an artificial with an authentic fragment. The length of both the authentic and artificial fragments was randomly varied between 2-4 lines. In total, 6,381 authentic fragments were presented to the users against 6,272 artificially generated rap fragments, which are evenly distributed over six model types (cf. Table 1, see below for a detailed account of these six models).

**Table 1:** Statistics on the number of stimuli.

	Conditional	Unconditional	Total
Character	912	1,335	2,247
Hierarchical	1,045	963	2,008
Syllable	1,038	979	2,017
**Total**	2,995	3,277	6,272

Stimuli are evenly distributed over the 6 (2 x 3) model types considered in this study.

### Data

The training data for our language generation models was derived from data collected from the *Original Hip-Hop (Rap) Lyrics Archive* (OHHLA) (http://www.ohhla.com/). OHHLA is an online archive documenting Hip-Hop lyrics since 1992. The archive contains over 60,000 transcriptions of rap lyrics, encompassing the main body of English Hip-Hop music produced in the United States of America. While other suitable online rap platforms exist (e.g. Genius), we chose OHHLA as it allows more efficient downloading of the lyrics, and shares many transcriptions with more well-known platforms. The downside of using lyrics from these online platforms is that it cannot be demonstrated to what extent quality control measures have been applied by the transcribers. However, since OHHLA (like Genius) is a community effort, with many active contributors commenting on and correcting rap transcriptions, we can be confident that the quality of the rap lyrics is good enough for our purposes. Data were downloaded from the online archive resulting in a corpus of 64,542 songs by 28,099 different artists (including artist collaborations and ‘featuring’ appearances of artists). Preprocessing of the data consisted of three subsequent steps. First, all songs were tokenized using the Ucto software (see https://languagemachines.github.io/ucto and corresponding manual https://github.com/LanguageMachines/ucto/raw/master/docs/ucto_manual.pdf). After tokenization, the total corpus comprised 37,236,248 tokens. In their lower-cased form, 380,013 of these word forms occur uniquely (cf. Table 2). On average, each song consists of approximately 576 words, and each artist is represented by 2.3 songs (cf. Table 3).

**Table 2:** Corpus statistics.

Songs	Artists	Words	Vocabulary
64,542	28,099	37,236,248	380,013

Counts of the total number of songs, artists and words collected from the *Original Hip-Hop (Rap) Lyrics Archive* (OHHLA).

**Table 3:** Song statistics.

Words/Song	Songs/Artist	Words/Artist
576.93 (± 223.77)	2.3 (± 7.65)	1325.2 (± 4223.2)

Statistics on the average number of (1) words per song, (2) songs per artist, and (3) words per artist.

To allow language generation on the level of syllables (see below), the second preprocessing step comprised the segmentation of all unique word forms (i.e. word types) into syllables. To this end, we developed a simple neural tagger, which was trained on the CMU Pronouncing Dictionary [30]. The tagger processes each word character by character and classifies each consecutive character as either a syllable boundary (1) or syllable internal character (0). Thus, a word such as *rapping* is analyzed as 1001000, which can easily be translated into a traditional syllable representation (i.e. *rap-ping*). For more information about this implementation, we refer to the code repository associated with this paper. The syllabified corpus consists of 43,531,133 syllable tokens, and a syllable vocabulary of 89,337.

In the third and final step, we translated all word types into a phonological representation, using the Grapheme-to-Phoneme (G2P) conversion toolkit [31] (see https://github.com/cmusphinx/g2p-seq2seq). The system implements a transformer learning mechanism, which has proven efficient to draw global dependencies between the input (the graphemic representation) and the output (the phonemic representation). In addition to sound representations, the phonological representation indicates which vowels have primary or secondary stress. This phonological representation enables us to conveniently extract pairs of rhyming words as discussed below.

### Language generation models

We generate text by sampling from a Language Model implemented on the basis of a Recurrent Neural Network. A Language Model is an estimator of the probability of any given sentence. Probabilistically, a Language Model decomposes the probability of a sentence into the product of the conditional probabilities of its parts (which can be words, characters, syllables, or any other segmentation of the input sentence) given the history. Eq 1 formalizes the given definition for a sentence of *n* words.

$$P\left(w_{1}\ldots w_{n}\right)=P\left(w_{1}\right)\cdot \prod_{t=2}^{n}P\left(w_{t}|w_{1}\ldots w_{t-1}\right)$$ (1)

Text generation with a Language Model is based on sampling from the output probability distribution and feeding the resulting sampled token back into the model to obtain a new probability distribution.

In order to estimate the conditional distribution, a Markovian assumption is traditionally made, which limits the conditional chain up to a fixed number of tokens. Alternatively, a Recurrent Neural Network can be used to avoid the fixed history limitation and incorporate distributional features into the estimation. In recent years, such Neural Language Models have shown excellent performance, holding the current state-of-the-art in Language Modeling (see e.g. [32]). A Neural Language Model is trained with Stochastic Gradient Descent to optimize the following objective:

$$P_{RNN}\left(w_{t}|w_{1}\ldots w_{t-1}\right)\propto exp\left(O\cdot h_{t}\right)$$ (2)

where *O* ∈ **R**^|*V*|×*d*^ is a projection matrix from the hidden dimensionality *d* onto the vocabulary size |*V*| and *h*_*t*_ corresponds to the hidden activation at step *t* computed by a Recurrent Neural Network: *RNN*(*v*_*t*_, *h*_*t*−1_). Algorithmically, an RNN implements a function that takes as input a vector *v*_*t*_ representing the input word at the current step and the previous hidden activation *h*_*t*−1_, thus facilitating theoretically unlimited left-to-right information flow during processing. While multiple definitions of the *RNN* function have been studied in the literature, LSTMs are a popular choice, with strong performance in Language Modeling [33]. An LSTM cell incorporates three learnable gates into the recursive mechanism (an input, forget and output gate) that are defined by the following Equations:

$$i_{t}=\sigma \left(W_{ih}^{i}v_{t}+W_{hh}^{i}h_{t-1}+b^{i}\right)$$ (3)

$$f_{t}=\sigma \left(W_{ih}^{f}v_{t}+W_{hh}^{f}h_{t-1}+b^{f}\right)$$ (4)

$$o_{t}=\sigma \left(W_{ih}^{o}v_{t}+W_{hh}^{o}h_{t-1}+b^{o}\right)$$ (5)

where $W_{ih}^{*}\in R^{dxH}$ and $W_{hh}^{*}\in R^{HxH}$ are, respectively, the input-to-hidden and hidden-to-hidden projection matrices of the corresponding gate and b* ∈ **H** is a gate-specific bias. Besides the gating mechanism, the LSTM incorporates a writable memory cell *c*_*t*_ that is updated following Eq 6:

$$c_{t}=f_{t}⊙c_{t-1}+i_{t}⊙\text{tanh}\left(W_{ih}^{c}v_{t}+W_{hh}^{c}h_{t-1}+b_{h}^{c}\right)$$ (6)

where ⊙ is element-wise product and tanh is the hyperbolic tangent non-linear function. Finally, the memory cell is combined with the vector coming from the output gate to yield the hidden activation *h*_*t*_: *h*_*t*_ = *o*_*t*_ ⊙ *σ*(*c*_*t*_), which can be used as an abstract representation of the sequence at that particular step.

#### Modeling levels

Language Modeling is typically done at either character or word level, each variant featuring its own characteristics. On the one hand, character-level modeling considerably reduces the vocabulary size (i.e. in the present corpus from 89,337 syllables to 172 characters), and expands the number of tokens in the data-set by a factor proportional to the average word length. (The seemingly large number of characters is the result of diacritics and punctuation marks, including alternative punctuation marks). On the other hand, word-level models require less input tokens to process larger text spans by a factor again proportional to the average word length, and thus may be able to better exploit long-term dependencies in the dataset.

In the present case, we train our models with both character and syllable-level objectives, and explore how the different design and modeling choices affect the realism of the produced text. The decision to compare character and syllable-level models instead of character and word-level models is rather atypical and deserves some explanation. The reasoning is twofold: on the one hand, the type-token ratio in user-generated content is artificially reduced due to noisy variation. In such a scenario, a word-level model has to deal with an exploding vocabulary size, and the evidence for a large proportion of types is very sparse. On the other hand, rap music adopts a particularly rhythmic style where syllables can be considered more central to text composition than words. As such, the syllable model is expected to perform better than the character-level model, because the latter is agnostic to word structure and must therefore rely on more general cues to reproduce the rhythmic style of rap. Thus, we consider the character-level model as the baseline model in this study. This is additionally justified given that purely count-based models are easily outperformed by RNN-based architectures in Language Modeling when working with sufficiently large datasets.

Besides purely character and syllable-level models, we also experiment with a hierarchical architecture. Since this architecture is relatively uncommon, we will explain its composition in greater detail. Our hierarchical model is similar to a word-level LM in that it decomposes the probability of a sentence into the conditional probabilities of its words but, additionally, decomposes the probability of each word on the basis of its constituent characters:

$$\begin{array}{c} \begin{array}{cc} P(w_{t}|w_{1}\ldots w_{t-1}) & =P(c_{1}\ldots c_{|w_{t}|}|w_{1}\ldots w_{t-1})\\ & =P\left(c_{1}|w_{1}\ldots w_{t-1}\right)\cdot \prod_{i=2}^{|w_{t}|}P\left(c_{i}|c_{1}\ldots c_{i-1};w_{1}\ldots w_{t-1}\right) \end{array} \end{array}$$ (7)

The probabilistic model defined by Eq 7 can be implemented with multiple RNN layers operating at different scales. At the input layer, words are processed as a series of character-level embeddings by a bidirectional RNN, extracting a single distributional feature vector for each word. In a second layer, the sequence of word embeddings is processed by a second RNN producing word-level hidden activations *h*_1_…*h*_*n*_. Finally, a third RNN is run, conditioned on the hidden activation *h*_*t*_, to estimate the next word’s probability in a character-by-character fashion.

While in the non-hierarchical case conditioning on the history is ensured by the recursive mechanism itself, in the hierarchical case we have to ensure conditioning across different scales. One possibility to accomplish this is to initialize the hidden state of the third RNN with *h*_*t*_. In the present case, we instead implement conditioning by concatenating *h*_*t*_ to each vector input to the third RNN. The main difference is that our approach provides more credit assignment paths (proportional to the average syllable length in the data-set), and allows different hidden dimensionalities at different layers in the network.

The hierarchical model has certain advantages that we expect to result in increased sample quality. Different layers are in charge of information flow at different scales, providing both the compactness of a character-level model and the capacity to extract longer-term dependencies typical of word-level models. Our hierarchical architecture can be considered a specific case of a general hierarchical multi-scale model [34], which additionally learns a segmentation model as a result of the training. Our model, however, is less general since it assumes fixed segmentations at syllable or word boundaries. Still, it achieves similar perplexity scores on the Penn Tree Bank benchmark corpus (cf. Table 4).

**Table 4:** Text generation model details.

Type	Conditional	#Parameters	Dev Perplexity
Character	✓	12,613,079	1.65
		12,966,517	1.55
Syllable	✓	29,835,804	46.12
		29,914,104	33.43
Hierarchical	✓	14,492,179	1.38
		14,913,879	1.27

In agreement with the literature, we report Perplexities for syllable-level models and bits per character (BPC) for character-level models.

#### Hybrid input embeddings

The explanation in the previous paragraphs concerns the more conventional hybrid approach, going from the character-level to the word-level. As outlined above, we apply the same methodology, but going from the character-level to the *syllable-level*: the hierarchical LM processes first the input at the character level and then at the syllable level. This allows us to enrich the input to the second layer with extra syllable-level embeddings. We do this by concatenating both the output of the character-level syllable embeddings and syllable embeddings coming from a dedicated embedding matrix. Similarly, in the case of the syllable-level LM, the syllable-level input embeddings can be enriched with an additional bidirectional RNN over characters. This is an optimization that helps us ameliorate the problem of exploding syllable-level vocabulary sizes without having to worry about too restricted embedding matrices (since we would still be able to compute a character-level embedding for out-of-vocabulary items).

#### Conditioning

The conditioning procedure described above can easily be extended to condition the generative process on specific sentential information, such as tense, mood, sentiment or style (formal vs informal) [3]. For a given set of categorical variables *C* = {*C*_1_…*C*_*m*_}, the basic probabilistic model for sentence *s* is shown in Eq 8:

$$P\left(w_{1}^{s}\ldots w_{n}^{s}\right)=P\left(w_{1}^{s}|C_{1}^{s}\ldots C_{m}^{s}\right)\cdot \prod_{t=2}^{n}P\left(w_{t}^{s}|w_{1}^{s}\ldots w_{t-1}^{s},C_{1}^{s}\ldots C_{m}^{s}\right)$$ (8)

where $C_{1}^{s}\ldots C_{m}^{s}$ refers to the value assignments for the set of conditions *C* for sentence *s*, already known at training time. Most importantly, such conditioning makes it possible to control for the values of the particular conditioning values during generation.

In the present study, we condition our models on sentence length and final sentence phonology (i.e. last stressed vowel and the phonology of any following syllable). As we show below, this conditioning allows us to enhance the realism of the generated rap lyrics by biasing the generative process towards particular verse structures and higher rhyme density.

#### Generation

**Temperature** Given a trained Language Model, texts can be generated by multinomially sampling from the output probability distribution at each step. However, in order to generate realistic samples, it is a common practice to skew or flatten the output distribution by dividing the logits with a factor *τ* known as “temperature”. (In the literature, the term “logits” is commonly used to refer to the raw prediction values produced by the output layer before normalization into a proper probability distribution by the softmax function.) This modification allows us to control a trade-off between the grammaticality and diversity of the output text. For the present study, *τ* was selected on a model-per-model basis by manual inspecting the model output at different temperature values and selecting the best value according to our own assessment of the output quality.

**Templates and rhyming** Given our conditioning scheme, we propose to enhance the realism of the generated text by extracting templates from the dataset and apply their underlying conditioning values to the generation of a new text. Therefore, in order to generate text, we first randomly sample a fragment from the training corpus and extract the lengths and final sentence phonology of each of the sentences in the fragment. Following the template, we generate as many sentences as there are in the sampled template using the extracted conditions. We restrict ourselves to snippets of lengths between 2 and 4 lines (cf. Table 5 for some examples of generated lyrics samples).

**Table 5:** Examples of generated samples.

Percentile	Model	Sample
25%-50%	S	*it was too pretty (do it again)*
	S+	hey! like that, i got it like that i can’t believe, that you have a man ***** you don’t wanna **** with us
	H+	*yeah, yeah, yeah westside, uptown, chi-town, chi-town handle my business, put your trust in me*
50%-75%	C	*you know i got it like that i’m bringin mine this is how i get down*
	C+	*from all that and i don’t know what to do*
	H	*pay attention to the masses i’m just tryin to do it by myself*
75%-100%	C+	are you in the city? (uh) uhh, listenin’ to the break of dawn medical is a revolution some of that **** is going on late y’all
	H	ladies and gentlemen i’m all about my ************* money hanging out the window, trying to get the dough retaliation, pay attention
	H+	*it’s on, it’s on, it’s on tony montana, call the cops it’s like that*

Generated samples from the experiment randomly extracted from different difficulty bins (e.g. 25%-50% refers to examples in the 25%-50% difficulty percentile according to a logistic classifier). Models correspond to character-level (C), syllable-level (S) and hierarchical (H). The trailing “+” indicates a conditional model. Non-PC words have been deliberately masked out with asterisks in these examples.

**Summary** In sum, the full generation process conforms to the following steps. First, a temperature value is sampled from a Gaussian distribution τ∼N(μ,0.075). Second, for conditioned models, we uniformly sample a template from the database. Third and finally, a snippet is generated using sampled *τ* and template (whenever it applies). Formally invalid snippets were discarded. For instance, syllable level models are trained on limited vocabularies by masking any out-of-vocabulary tokens with a reserved symbol. During generation it is always possible that the reserved unknown symbol is sampled, which leads to a failed generation attempt. Additionally, we also filter out consecutive sentences that rhyme on the same word.

### Methods of evaluation

#### User response analysis

The user responses are analyzed using a series of logistic regression experiments from two main perspectives. First, we analyze whether participants can distinguish between authentic and artificial rap lyrics. In this setup, the dependent variable is whether the responses of the participants are correct or incorrect. As a complementary perspective, we analyze the authenticity of rap fragments as *perceived* by the participants, irrespective of whether their responses are correct or not. The analysis of perceived authenticity aids our understanding of possible perception biases towards artificial or original fragments. We analyze the effects of (i) trial number, (ii) generation model type (i.e. Character, Syllable or Hierarchical), and (iii) conditioned models. All experiments employ ‘participant’ as a variable intercept. The Logistic Regression models were fitted using the R package *brms* [35], which employs the ‘No U-Turn Sampler’ (NUTS) for sampling [36]. Priors for the main effects take the form of weakly informative normal distributions with zero mean a scale of 2. Intercept priors were set as normally distributed with a mean of zero and standard deviation of 5. Varying intercepts were modeled using a Half-Cauchy with zero mean and scale 1. All regression models were executed with four chains (2,000 iterations per chain of which the first half constitute warm-up iterations). Bayesian regression models do not report a single, most likely coefficient *β* but a distribution of *β* values. In the analyses below, we report on the mean, median and 95% Credible Interval (CI_95_) of this distribution. All models were assessed for convergence by evaluating their trace plots, as well as the $\hat{R}$ statistic [37]. Since all $\hat{R}$ values were confidently below 1.1 (indicating good convergence), we refrain from reporting individual values. When informative, we evaluate and compare the regression models’ performance on out-of-sample data using the Widely Applicable Information Criterion (WAIC) [38].

#### Linguistic features

To further examine possible motivations behind the participants’ authenticity judgments, we perform a linguistic feature analysis to highlight potential linguistic characteristics which participants *associate* with or *perceive* as generated or authentic materials. We analyze the effect of a broad range of features, which were selected on the basis of (i) prior research describing linguistic properties of generated text [22, 23], and (ii) research describing characteristic properties of rap lyrics [15, 16, 18, 19].

- **Prosodic Features**
  - *Alliteration*: Alliteration is defined as successive words with identical initial consonant sounds. We measure the fraction of alliterating words per fragment. Similar to rhyme density (see below), alliteration is considered to be a key prosodic feature of rap lyrics.
  - *Assonance*: The assonance score aims to capture resemblances of sounds between stressed syllables. We compute the assonance score as the fraction of words sharing the most common stressed nucleus in a generated or authentic rap fragment.
  - *Rhyme Density*: Rhyme density is measured as the fraction of successive line-final words with identical stressed vowels. This liberal definition of rhyme allows us to incorporate and approximate the broad spectrum of rhyme (e.g. slant rhyme) often observed in rap.
- **Morphological Features**
  - *Word Length*: As an approximation of word and morphological complexity, the average word length in number of syllables was computed for each generated and authentic rap fragment.
- **Syntactic Features**
  - *Mean Tree Span*: The Mean Tree Span score is one of the two features measuring the syntactic complexity of rap lines. It refers to the average length in constituents spanned by a non-terminal node in the dependency tree.
  - *Mean Tree Depth*: The Mean Tree Depth score is a second measure approximating the syntactic complexity of rap lines. It refers to the average depth of each subtree in a dependency tree.
- **Lexical Features**
  - *Non-PC Vocabulary*: During the experiments, it was repeatedly noted by the participants that the usage of politically incorrect vocabulary could be a feature of original, authentic material. We measure the fraction of words in a rap fragment that are part of a list of typical politically incorrect words.
  - *Lexical Diversity*: A well-known characteristic of neurally generated text is its relatively low lexical diversity. We measure the lexical diversity of rap fragments by computing their Shannon Entropy.
  - *Word Repetition*: Captures the amount of successively repeated words, which is a known artifact of RNN-based text generation systems. We measure it as the fraction of identical successive words in each fragment.

Table 6 provides a statistical overview of these nine features. The table shows the mean and standard deviation values for each of the nine features for both generated and original fragments. We fit and compare three binomial logistic regression analyses. First, we analyze to what extent these features are helpful in objectively distinguishing generated from authentic materials. Second, we analyze the effect of the linguistic features on the participants’ perception of authenticity, taking their response (i.e. “generated” or “authentic”) as the dependent variable. Third and finally, we zoom in on a small group of high-scoring participants, to test how the presence or absence of certain linguistic features affects their judgment behavior.

**Table 6:** Linguistic feature statistics.

	Generated						Authentic	
	Character		Hierarchical		Syllable			
Alliteration	0.045	(0.057)	0.05	(0.063)	0.046	(0.057)	0.044	(0.047)
Assonance	0.14	(0.074)	0.16	(0.076)	0.13	(0.068)	0.14	(0.058)
Rhyme density	0.1	(0.26)	0.11	(0.27)	0.12	(0.27)	0.21	(0.35)
Word length	1	(0.0041)	1.4	(0.23)	1.2	(0.14)	1.2	(0.15)
Mean tree span	2.4	(0.77)	2.6	(0.84)	2.6	(0.88)	2.4	(0.57)
Mean tree depth	2.2	(0.28)	2.2	(0.28)	2.2	(0.26)	2.7	(0.39)
Non-PC vocabulary	0.025	(0.043)	0.038	(0.055)	0.028	(0.049)	0.022	(0.038)
Lexical diversity	2.6	(0.37)	2.5	(0.33)	2.6	(0.37)	3	(0.39)
Word repetition	0.006	(0.038)	0.009	(0.05)	0.006	(0.033)	0.002	(0.01)

Statistics (mean and standard deviation) for the presence of linguistic features in fragments generated by the three types of conditioned language models. The final column includes the same statistics for authentic rap fragments.

## Results

### Objective authenticity

We first address the question whether participants could correctly distinguish between original and generated lyrics, i.e. *objective authenticity* (RQ1). On average, authenticity detection accuracy was above chance level (50%), with participants correctly distinguishing between original and generated text in 61.1% of the time (corresponding to an odds ratio of 1.57, 95% credible interval (CI_95_): [1.48, 1.66]). With a 58% median accuracy (CI_95_: [56.3%, 60.1%]), participants performed significantly worse on questions of type-B than of type-A (64%; CI_95_: [61.9%, 65.5%]), suggesting that the task becomes harder in the absence of a reference point.

**Fig 2:**
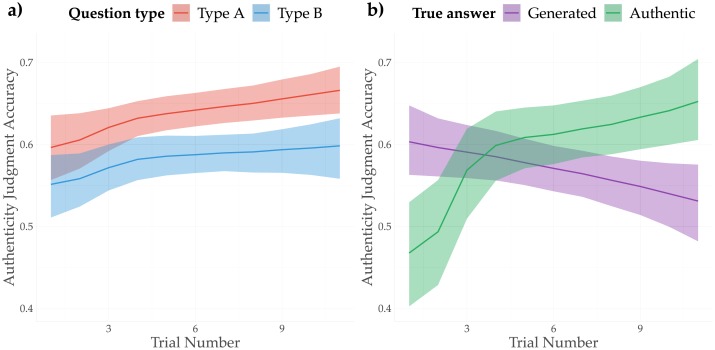
Marginal effects plot of trial number on the authentication accuracy. **A**: Marginal effects plot of trial number for both type-A and type-B questions; **B**: Marginal effects plot showing the effect of trial number on the accuracy of classifying text fragments as “Authentic” or “Generated” in type-B questions alone.

There is mild evidence for the presence of a learning effect influencing the authenticity detection accuracy as participants progressed through the game (OR: 1.02; CI_95_: [1.01, 1.04]). We model the effect of trial number as a monotonic effect, which allows us to efficiently estimate predictors on an ordinal scale [39]. The coefficients (and corresponding odds ratios) are to be interpreted as the average difference between adjacent time steps. As can be observed from the marginal effects plot in Fig 2a, the effect is present in both question types, and it is most pronounced at the beginning of the game, after which it diminishes. Importantly, however, the presence of a possible learning effect should be explained differently for the two question types. In type-A questions, participants had to choose which of the two presented fragments was authentic, whereas in type-B questions they had to judge the authenticity of a *single* fragment. Thus, by design, the learning effect for type-A questions can only involve the increasing accuracy of selecting the original fragment. Importantly, however, while for type-B questions the learning effect also involves an increasing precision of detecting *authentic lyrics*, it does *not* positively affect the accuracy of spotting generated lyrics. A separate logistic regression for type-B questions confirms this interaction (OR: 1.11; CI_95_: [1.06, 1.16]). This is illustrated by the marginal effects plot in Fig 2b. In fact, the slightly negative slope of the accuracy of spotting generated lyrics over time counters the hypothesis of a learning effect in type-B questions. As will be argued in more detail below, the difference between the two slopes is more likely to be attributed to a gradual change in perception bias than to a learning effect: initially, participants have a rather outspoken bias towards responding “Generated”, which is rapidly corrected for a bias towards “Authentic”.

### Perceived authenticity

**Fig 3:**
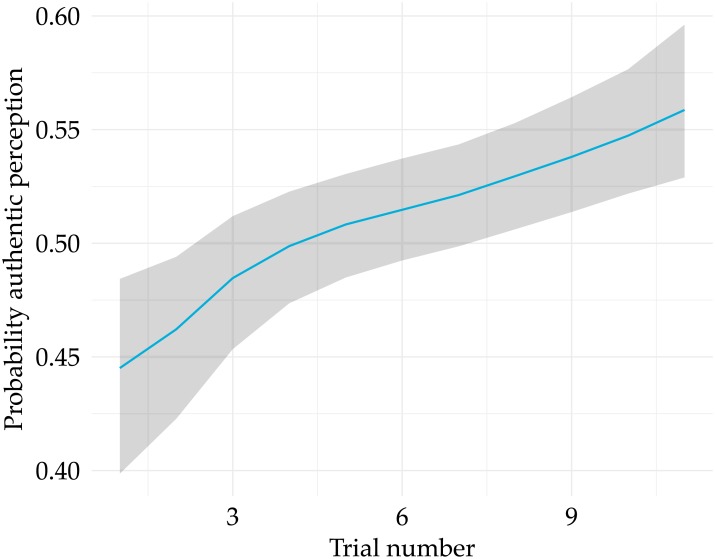
Marginal effects plot, showing the effect of Trial number on the *perceived* authenticity of text fragments.

By focusing on the authenticity detection accuracy, we ignore possible biases towards classifying text fragments as either ‘original’ or ‘generated’. To account for such biases, we turn to our second research question (RQ2), which concerns the probability of participants responding ‘original’ or ‘generated’—i.e. the *perceived* authenticity. In this analysis and the analyses below, we focus on type-B questions only, because in the case of type-A questions, we cannot rule out the possibility that judgments were made solely on the basis of the original text fragment (i.e. irrespective of the artificially generated lyrics). The overall probability of responding ‘original’ is approximately 50.98% (CI_95_: [49.4%, 52.6%]), suggesting the absence of a bias towards perceiving text fragments as generated. However, as briefly suggested above, the perception bias is not stable over time, and gradually moves towards perceiving fragments as ‘original’ (OR: 1.05; CI_95_: [1.02, 1.07]). Fig 3 displays the marginal effects of trial number on the perceived authenticity of text fragments. It is clear that while participants lean towards responding ‘generated’ at the beginning of the game (with a posterior probability of responding ‘original’ of approximately 44.4%; CI_95_: [39.9%, 48.4%]), they tend to judge lyrics as ‘original’ as they progress through the game (the probability of judging lyrics to be ‘original’ is approximately 55.9% and the end of the game; CI_95_: [52.9%, 59.6%] at the end of the game). This change in perception provides a better explanation of the negative slope for ‘Generated’ examples in Fig 2b: with an increasing bias towards ‘Authentic’, the accuracy for detecting ‘Authentic’ fragments will logically improve, but this same bias *negatively* affects the accuracy for detecting ‘Generated’ fragments.

### Model comparison

We continue with our third research question (RQ3) asking whether authenticity detection performance varied across language models and architectures. The performance of all models is above chance, with an accuracy of approximately 61.5% for the character level model (CI_95_: [57.5%, 65.4%]), 55.8% for the syllable level model (CI_95_: [51.5%, 60.1%]), and 53.1% for the hierarchical model (CI_95_: [48.7%, 57.4%]). Questions containing fragments generated by the syllable model had 0.79 (CI_95_: [0.63, 0.99]) times the odds of being correctly answered compared to questions with character level fragments. The proportion of posterior samples with a log-odds below zero (*θ* < 0) equals approximately 0.98 (evidence ratio (ER): 50.28), suggesting indeed sufficient evidence that participants performed worse when presented with syllable-level fragments compared to character-level generations. Hierarchically generated fragments were markedly harder to detect than character level fragments (OR: 0.71; CI_95_: [0.55, 0.9]), with a posterior probability of 1 (ER: 399) for a negative effect on the participants’ detection performance.

**Fig 4:**
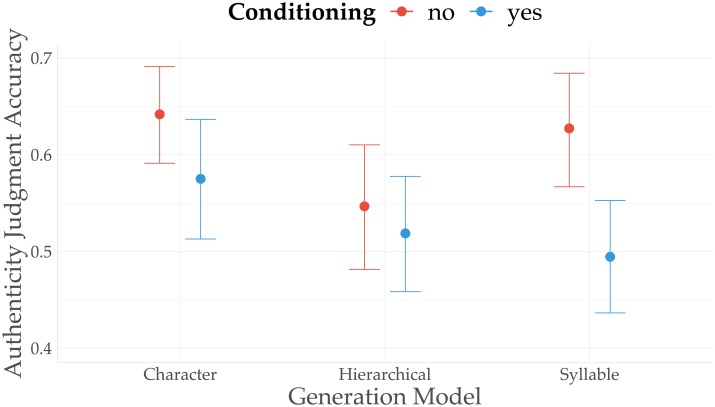
Marginal effects plot showing the effect of different language models. Marginal effects plot showing the effect of different language models in interaction with conditioning on the accuracy of classifying text fragments as “Authentic” or “Generated”.

It is interesting to compare the effects of the different language models with the effect of conditioned language generation. First, an analysis of the effect of ‘conditioning’ (irrespective of specific language models) reveals a clear decrease in authenticity accuracy when participants were presented with conditionally generated text fragments (OR: 0.7; CI_95_: [0.58, 0.85]). The mean accuracy drops from 61% (CI_95_: [57.7%, 64.5%]) for unconditionally generated lyrics to 52.6% (CI_95_: [49.1%, 56.2%]) when lyrics are generated using conditions. Second, while conditioning has a significant effect on authenticity detection accuracy overall, its effect varies across language models. As shown in Fig 4, the effect of conditioning is most clearly manifested in fragments generated with the syllable-level model, where conditioning lowers the authenticity detection accuracy from 62.7% (CI_95_: [56.7%, 68.4%]) to 49.5% (CI_95_: [43.7%, 55.3%]). Similarly, conditioning negatively impacts the detection accuracy for character-level generations (unconditional: 64.2%; CI_95_: [59.1%, 69.1%]; conditional: 57.5%, CI_95_: [51.3%, 63.7%]). Finally, we observe a mild effect of conditioning on hierarchically generated text fragments, with a mean accuracy of 54.7% (CI_95_: [48.2%, 61%]) for unconditioned generations, and a mean accuracy of 51.9% (CI_95_: [45.9%, 57.8%]) for conditioned generations.

To better understand the effects of conditioned language generation, different language models and their interaction, we compare their out-of-sample deviance by means of the Widely Applicable Information Criterion (WAIC). Table 7 presents (i) the WAIC scores of the different models, (ii) the difference between each model’s WAIC and the best WAIC, (iii) the standard error of these differences, and (iv) the Akaike weight of each model. The regression model which analyzes the interaction between generation level and conditioning has the lowest WAIC score, and weighs 77%. Note, however, that the interaction model is hardly distinguishable from the model which only tests for the effect of conditioned language generation. The difference between the two WAIC scores, *δ*_WAIC_, is only 2.53, which is much smaller than the standard error of the difference (*δ*_SE_ = 6.12). The top two models are both better than the model with only generation-level as population-level effect. This suggests that conditioned language generation in particular has a strong effect on the participants’ guessing behavior, and that different language models have a stronger effect in interaction with conditioned language generation.

**Table 7:** WAIC scores as estimates of out-of-sample deviance.

	WAIC	*δ*_WAIC_	*δ*_SE_	weight
language model * conditioned	2488.5	0	0	0.767
conditioned	2491.1	2.53	6.12	0.216
language model	2496.2	7.65	7.31	0.016

Table showing (i) the WAIC scores of the different regression models, (ii) the difference between each model’s WAIC and the best WAIC, (iii) the standard error of these differences, and (iv) the Akaike weight of each model.

### Linguistic feature analysis

Our fourth and final question is concerned with the textual and linguistic features distinguishing original from generated text (RQ4). We will address this question from two angles. First, we investigate which linguistic features objectively distinguish original from generated text. As a second, complementary perspective, we examine which features contribute to the participants’ *perceived* authenticity. Comparing those perspectives (i.e. objective and perceived feature importance) allows us to zoom in on linguistic features underlying mistakes in authenticity judgments.

#### Objective feature importance

**Fig 5:**
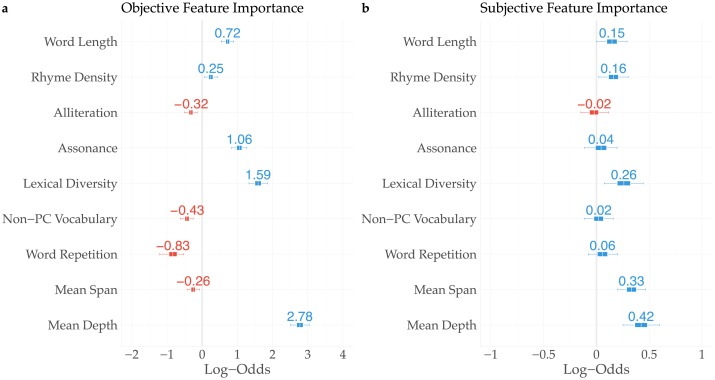
Estimates of objective (a) and subjective (b) feature importance.

To estimate objective feature importance, we perform a logistic regression analysis with as dependent variable whether a text is either original or generated, and as predictors all previously discussed linguistic features. These predictors were scaled using the R package *standardize* [40] to have zero mean and scale 0.5. As illustrated by the log odds in Fig 5a, original and generated fragments differ with respect to a considerable number of linguistic features. First, with a Log Odds (LO) of 2.78 (CI_95_: [2.51, 3.04]) in favor of original text fragments, the average depth of syntax trees catches the eye. This tells us that, on average, generated text fragments make use of considerably less complex sentence constructions. Note however that the second feature approximating syntactic complexity, ‘Mean span’, appears not to have a positive effect, possibly due to the fact that it correlates heavily with the mean depth of syntax trees. A second and similar effect is visible in ‘Word Length’, computed as the average number of syllables per word in a fragment. The positive estimate of 0.72 suggests that morphologically more complex words are positively correlated with original lyrics (CI_95_: [0.54, 0.9]). A third strong predictor of authenticity is concerned with the ‘Lexical diversity’ of the lyrics. The relatively high estimate of 1.59 (CI_95_: [1.33, 1.86]) clearly shows the more diverse nature of authentic lyrics in terms of their vocabulary. A feature related to ‘Lexical diversity’ is ‘Word repetition’, which displays a negative mean estimate of -0.83 (CI_95_: [-1.2, -0.53]). As was expected, it shows that, on average, generated lyrics consist of a larger number of identical successive words. Of course, authentic lyrics also frequently employ repetition of words. However, the strong negative estimate suggests that the language generation models exaggerate this characteristic considerably. Turning to the stylistic features typically associated with rap lyrics, we observe that authentic lyrics are characterized by (i) a higher degree of vowel harmony (‘Assonance’ LO: 1.06; CI_95_: [0.84, 1.27]), and (ii) a higher ‘Rhyme density’ (LO: 0.25, CI_95_: [0.07, 0.43]). Interestingly, the amount of ‘Alliteration’ is markedly higher in generated lyrics than in authentic ones. The negative estimate of -0.32 (CI_95_: [-0.5, -0.14]) might indicate that the language generation models have overfitted on the presence of alliterating sequences in rap lyrics. Finally, we observe a pronounced negative estimate for the usage of politically incorrect or sensitive vocabulary. Again, the negative estimate of -0.43 (CI_95_: [-0.62, -0.26]) suggests that the language models have overfitted the highly frequent usage of such sensitive words.

#### Subjective feature importance

To estimate subjective feature importance, we repeat a similar procedure, in which the same linguistic predictors are used to model the perceived authenticity of text fragments. As can be observed from Fig 5b, a number of objectively important features are also subjectively deemed important to spot original text fragments. First, similar to the objective feature importance estimations, the average depth of syntax trees (LO: 0.42; CI_95_: [0.25, 0.6]) as well as the lexical diversity (LO: 0.26; CI_95_: [0.07, 0.44]) are two strong predictors, albeit their predictive power is lower. Second, participants also appear to observe the higher morphological complexity of original lyrics (‘Word Length’ LO: 0.15; CI_95_: 0.01, 0.29]), and increased density of rhyme words (LO: 0.16; CI_95_: [0.02, 0.3]).

Among the differences between objectively and subjectively salient features are (i) the mean span of syntax trees, (ii) repeated words, (iii) vowel assonance, (iv) alliteration, and (v) the fraction of politically incorrect words. First, with a Log-Odds of 0.33 (CI_95_: [0.20, 0.47]) in favor of ‘original’ text fragments, lyrics with larger ‘Mean Tree Spans’ were significantly more likely to be perceived as real. By contrast, the objective feature importance estimate in Fig 5a indicates an opposite, *negative* effect of Mean Tree Span, or, conversely, a preference for smaller values in original lyrics. A second notable difference is the lack of an effect of ‘Word repetition’. While generated lyrics often exhibit higher amounts of identical successive words, participants appear to be undecided as to whether this is a feature of generated or authentic lyrics (LO: 0.06; CI_95_: [-0.08, 0.2]). Third, neither ‘Alliteration’ (LO: -0.02; CI_95_: [-0.15, 0.11]) nor ‘Assonance’ (LO: 0.04; CI_95_: [-0.11, 0.2]) have a clear effect on the perceived authenticity, while objectively they represent relatively strong predictors. Finally, while objectively a feature of generated fragments, the occurrence of politically incorrect words does not seem to have affected the judgment of participants (LO: 0.02; CI_95_: [-0.11, 0.16]).

**Fig 6:**
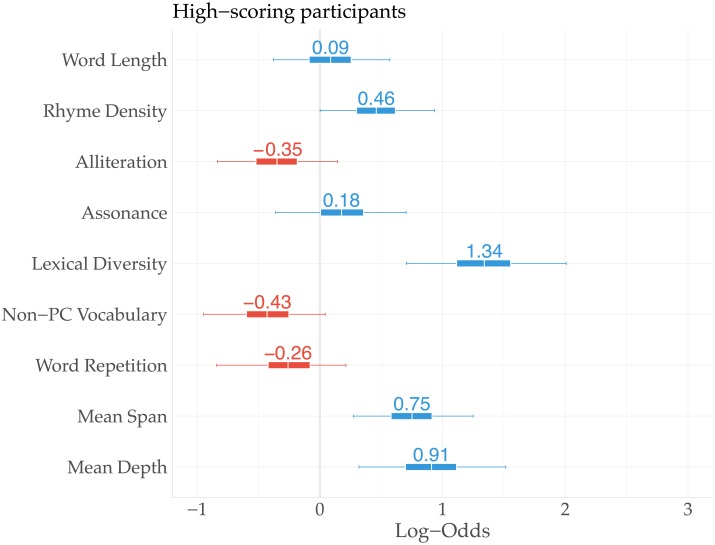
Estimates of (subjective) feature importance for high-scoring participants.

We conclude this section with an analysis separating high-scoring participants from the others. High-scoring participants are defined as those with a score higher than one standard deviation from the mean score. This amounts to 72 participants with a score higher than 12. The same set of linguistic features is used to fit a logistic regression model for this group of players. Fig 6 presents the mean estimates. Overall, the results are slightly more pronounced, and the perceived feature importance seems more in line with the objective feature importance (cf. Fig 5a). For example, ‘Mean Depth’ of syntax trees (LO: 0.91; CI_95_: [0.32, 1.52]), ‘Lexical Diversity’ (LO: 1.34; CI_95_: [0.71, 2.01]), and ‘Rhyme Density’ (LO: 0.46; CI_95_: [0, 0.93]) function as much stronger indications of authenticity for high-scoring participants. Furthermore, while ‘Word Repetition’ previously barely affected authenticity perception, high-scoring participants are more likely to consider repetitiveness a key characteristic of artificially generated lyrics, which is also objectively correct. Finally, high-scoring participants seem to be more aware of the unusual amount of politically incorrect or otherwise sensitive words generated by the language models (LO: -0.43; CI_95_: [-0.95, 0.04]; posterior probability of a negative estimate: 0.98; ER: 24.48).

## Discussion

While natural language generation has witnessed impressive advancements in recent years, the evaluation of such generative systems remains a difficult issue. In this paper, we have discussed a methodology of estimating the quality of a number of neural language generation systems and configurations, through crowd-sourced authenticity judgments of artificially generated rap lyrics. In this final discussion, we wish to offer some additional reflection on and contextualization of our findings, by reviewing our four research questions one by one.

### RQ1: *To what extent can participants correctly discriminate between original and generated lyrics*, *i*.*e*. objective authenticity

After controlling for a number of confounding effects, we found that the participants’ performance to distinguish authentic rap lyrics from artificially generated lyrics was only marginally above chance. Thus, it appears that, in many cases, the generations systems were able to successfully ‘deceive’ the participants. However, we should note two important caveats. First, our setup works with relatively short fragments (2-4 lines). Insights from previous studies suggest that for longer fragments the artificial fragments would exhibit a lack of semantic coherence, which would make the participants’ discrimination task easier [23]. Second, some caution is required in interpreting the results showing that type-B questions, where no point of authentic reference was available, were notably harder to judge. This suggests that the participants’ task was generally easier when the authenticity judgment task was framed in a relative way. However, in many instances type-B questions (generated or not) consisted of bleak, highly formulaic fragments that were too short to contain strong cues regarding their authenticity. It should be noted, therefore, that the observed effect might also be reduced when longer fragments are admitted, which is something for future research to reveal.

### RQ2: *How is the authenticity of rap lyrics perceived by the participants*, *i*.*e*. *irrespective of whether their answer is objectively correct?*

Perception bias plays an important role in the way we tend to interpret the observed learning effects in the experiments. Given that the participants were informally and emphatically informed that they would see non-authentic rap, it seems unsurprising that participants were initially biased towards perceiving fragments as generated. This kind of ‘onset suspicion’ could be interpreted as an urge among the participants to demonstrate that they could not be ‘fooled’ by a computer. In terms of social capital, the reward for not being deceived by a generated fragment seemed larger than the penalty for missing an authentic fragment. Especially for type-B questions, the bulk of the learning effect can be attributed to the gradual reduction of this bias towards the perception of texts as being generated. This effect might be a result of the immediate, visual feedback which participants received on the screen after answering each question. Thus, in the future, it might be worthwhile to re-run a similar analysis without giving immediate feedback, to verify whether any learning effects would still be present. Interestingly, most of the learning happens during the initial two or three questions, and the effect quickly stagnates after this. The perception bias found here ties in with prior text generation perception research [26, 29], in which similar test-initial biases towards generated material are reported. Overall, the shift in bias could be viewed as an act of normalization on the participants’ part: initially, many of them seem to have under-estimated the qualities of generated texts and over-estimated the quality of authentic texts. It only took them a two to three trials to realize that generated and authentic text were less distinct than they originally anticipated.

### RQ3: *Does the participants’ authenticity detection performance vary across different language generation architectures?*

Overall, computational linguists will be pleased to observe the strong effect the generation model type appeared to have on the quality of the text architecture. The type of model behind a generation and, crucially, the language unit at which text is generated (e.g. character or syllable) matters and has a significant impact on the authenticity judgments, suggesting that text realism can be improved through architectural innovations irrespective of whether such innovations are accompanied by improvements in customary Language Modeling evaluation benchmarks. However, conditioned language generation appears to be even more important, and its presence or absence was shown to be more important in explaining the user judgments. Conditioning generally helps all models: with conditioning, the judgment accuracy moves even closer to a chance-level accuracy. This could be expected in many ways, as conditioning implies the direct injection of hand-crafted, domain-specific knowledge about the poetics of the genre. Still, the importance of conditioning is not a trivial finding and ties in with a long history in template-based Natural Language Generation research [1]. The positive effect of conditioning shows that even with relatively superficial domain knowledge, clear improvements can be achieved. As such, we believe that, in addition to developing new and better text generation architectures, developing ways to better integrate such domain knowledge is also a fruitful path for future research [41, 42]. A final important and novel finding was the interaction between the generation model type and the presence or absence of conditioning in the model. The syllable-level model most impressively benefited from the inclusion of conditions. This can possibly be explained by the fact that most of the constraints encoded in the conditions are properties that manifest themselves at the syllable-level (e.g. rhyme patterns). That makes it easier for the model to make direct associations between the input and output of the networks.

### RQ4: *Which textual and linguistic features distinguish authentic from generated text*, *either objectively or subjectively?*

Concerning the feature analyses, there are several interesting observations to be made. The comparison of the objective and subjective results suggests that human classifiers sharply differ from the automated classifiers regarding which individual feature types they devote cognitive attention to (and the extent to which they do so). In other words, participants were unable to exploit the readily available linguistic differences to guide their authenticity judgments. Humans (often unsuccessfully) assign very different interpretations to several feature types which the automated classifiers were able to capitalize on (more successfully). The classifier, for instance, recognizes that generated text over-prefers the use of sensitive language (non-PC words), whereas the presence of such vocabulary for humans only functions as a mild cue to perceive a fragment as real. A striking parallel finding was reported in the context of text generation systems producing Adult content [43], which also generate sensitive or otherwise strong language, and deceive participants more easily than systems producing language in other domains. It remains an interesting question for further research how these results relate to our findings, and whether they are exemplary of a more general bias to perceive sensitive and strong language as more likely human than artificial. Besides lexical material, features relating to syntactic complexity and lexical repetition in particular show marked differences, the clear and evident take-home message here being that the more complex language a system produces, the less likely it is to be detected as non-authentic. However, we again wish to call for some caution as to not over-interpret the results, since the current study only covered a limited set of linguistic features. Future work should be directed toward understanding the effect of other, higher order features, such as the agreement of anaphora, semantic coherence, and topic, which can be extracted more easily and reliably for longer texts.

## Conclusion

Natural language generation offers increasingly valuable possibilities for real-world applications in the industry, as well as the creative arts. As techniques continue to mature in this field, we can expect a further intensification, in various constellations, of the contact between human language users and systems for automated text generation. The perceived authenticity of such systems—be it in a conversational setting or not—will be key to these systems’ success: in many domains, we can expect a more meaningful engagement with a system on the user’s part, if the system features human-like behaviour. While we do not expect new rap songs penned by artificial ghostwriters any day soon, prior research has shown the value of collaborative NLG systems, in which humans are assisted by NLG systems in their writing process [21, 44, 45]. The experiments reported above show that for short textual fragments, restricted to a focused domain, encouraging results can be obtained for various model architectures. As such, the systems developed in this study might prove useful in such collaborative contexts. While plain language models achieved a more than reasonable performance, the injection of hand-crafted domain knowledge via conditioning had a remarkable boosting effect on the ability to deceive the participants. Quite evidently, the benefits of the template-based conditioning approach presented in the current study extend beyond the generation of rap lyrics to other forms of lyrical content, such as pop song lyrics and poetry [46], and domain-specific language generation in general. Since conditioning seems to have a greater performance impact than different model architectures, more research effort should be directed to efficiently and flexibly integrate domain-specific data models into (neural) language generation systems. It should be stressed, however, that the limited length of the fragments used in our experiments makes it doubtful to what extent our findings will generalize to a more realistic setting, where the authenticity of longer fragments would be at stake. Finally, an intriguing aspect of our study concerned the observation of complex learning effects, which suggested that participants gradually learned to correct their initial biases—likely resulting from a social urge not be ‘fooled’ by a machine. This phenomenon of ‘onset suspicion’ on the user side, reminds us of the plain fact that communicative situations are a social given, just as much as they are a linguistic given.
